# Works on Pathological Anatomy

**Published:** 1835-07-01

**Authors:** 


### WORKS ON PATHOLOGICAL ANATOMY.

1. ILLUSTRATIONS OF THE ELEMENTARY FORMS OF DISEASE. By *Robert Carswell*, M.D. Professor of Pathological Anatomy in the University of London, &c. Fasciculus Seventh—Mortification. Four coloured Lithographic Plates.

2. ANATOMIE PATHOLOGIQUE DU CORPS HUMAIN, &c. Par *J. Cruveilhier*. Maladies du Fœtus. Livraisons XV., XVI., XVII., XIX.

WE do not associate these works, because there is any natural connexion between mortification and the various diseases of the fœtus, but simply because they are both in process of publication at present, and both deserve to be brought before the profession. Of pathological anatomy we have spoken so often, and said so much, that it would be worse than tautology to utter another word upon the subject. But, taking the view we do of the value of an extensive acquaintance with its facts, we feel ourselves compelled to allow no opportunity of laying them before our readers to escape us.

We shall first present an account of the fasciculus of Dr. Carswell. It is dedicated to the subject of Mortification, which is handled with much vigour and precision.

Dr. C. first points out the ordinary distinctions, as known and adopted in this country, between gangrene and sphacelus—dry and humid gangrene, and gangrena senilis—and so on. But he treats, in the fasciculus before us, of those states only which are usually comprehended under the term mortification. The several kinds of mortification he arranges under the three following heads:

1. Mortification from cessation of the circulation.
2. Mortification from the violent operation of mechanical, chemical, and physical agents.
3. Mortification from the deleterious influence of certain poisons.

For nosological arrangements of this sort we have usually but small respect. For all that they can do, is to furnish a more convenient method of expressing a certain number of facts, and, provided that they do this, their systematic merits or demerits may be disregarded. But to proceed. The first division is—

Mortification from Cessation of the Circulation.

Cessation of the circulation, says Dr. Carswell, in a part of the body may be produced,—1st, by inflammation; 2d, by mechanical causes, which obstruct the passage of the blood; and, 3d, by local or general debility.

1. *Mortification from Inflammation.* Mortification may occur as a result of inflammation in any tissue or organ of the body. The following sentiments require, perhaps, some consideration, before we can yield them implicit consent.

"Mortification is, however, much more frequently observed in those organs in which the vascular system predominates, or in which an inordinate accumulation of blood is readily produced, on account of their greater sensibility and their direct exposure to the influence of those causes which give rise to inflammation. Hence the reason why gangrene and sphacelus occur more frequently in the skin and cellular tissue, mucous membranes, and lungs, than in the other tissues and organs of the body, as immediate effects of inflammation; and why they are so rarely observed in serous and fibrous tissues, which contain few or no bloodvessels. Not only is mortification rarely observed in these latter tissues, but it may also be said never to occur in them as an immediate effect of inflammation, for they are never found in a state of gangrene or sphacelus, unless the cellular tissue with which they are in contact, and from whose vascular system their nutrition is derived, has previously been diseased. Such also is the case in caries (death of bone), as a consequence of inflammation of the periosteum and medullary membrane.

These circumstances enable us to explain why, in many cases, mortification takes place in one tissue, and not in another, although the inflammation by which it is preceded is the same in kind, degree, and duration. There are, however, many other circumstances of perhaps still greater importance, the single or conjoint operation of which favours, in a most remarkable manner, the termination of inflammation in gangrene and sphacelus: such as a state of chronic inflammation of a portion of an organ accompanied by induration and obstructed circulation; a state of local congestion, depending on the presence of an obstacle to the return of the venous blood: that state of general debility which prevails at the termination of protracted fevers, or during the first period of convalescence; and a morbid condition of the blood, such, for example, as that which occurs in scorbutus."

We do not think that the preceding observations are strictly accurate.

Dr. Carswell appears to insist on the pathological principle, that the greater the vascularity of a tissue, the greater the disposition to mortification. Now let us take the case of the cellular tissue and the skin. The vascularity of the former is certainly much less than that of the latter, yet its proneness to mortification is infinitely greater. We observe this in a remarkable degree in cases of diffuse inflammation of the subcutaneous cellular tissue after injuries. That tissue inflames and sloughs, while the skin (almost equally involved in the affection) continues to retain its vitality. In other cases, we see the inter-muscular cellular texture in a state of mortification, while the muscles, almost insulated by sloughs, are still alive. Now the cellular tissue is by no means vascular, yet it, of all others, is most prone to mortification. We might dwell more at length upon this subject, and adduce some further illustrations of the fact, that textures indifferently supplied with blood are very liable to inflammation and sloughing. General principles should always be considered with extreme circumspection before they are laid down, and received with much suspicion when they are proposed. But to proceed. Dr. Carswell next describes the general phenomena of gangrene and sphacelus, the more remarkable changes which take place in the circulation, innervation, temperature, colour, and consistence of the parts affected. These are best observed, and are consequently best described, as they appear in the skin and the subjacent textures. Yet Dr. Carswell does and can do no more than offer the usual epitome of the characters of mortification, contained in lectures and works of an elementary description. Such an epitome we may usefully omit, and pass to his remarks on Mortification of particular Tissues from Inflammation.

Mortification of the cellular tissue is considered first, and a knowledge of the characters and progress of this affection is highly important, indeed necessary to the surgeon. The cellular tissue, as we have already said, is, perhaps, more frequently the seat of mortification than any other texture, and displays the effects of that destructive process, in cases of "erysipelas phlegmonodes," on an extensive scale. As an acquaintance with the pathology of this frequent and very formidable malady is a matter of some consequence, we shall pause to consider it at some little length. Let us first introduce Dr. Carswell's description of the condition of a limb affected with the disease.

"When we examine a limb in which erysipelas phlegmonodes has gone through its several stages, we find that, in the first stage, the cellular tissue situated most remote from the original seat of the disease, is discoloured by the presence of minute vessels distended with dark blood, and contains a considerable quantity of serosity, which is sometimes nearly limpid. As we proceed nearer to the original seat of the inflammation, the quantity of the effused fluids increases to such a degree that the cellular tissue in which they are contained appears, from the great augmentation which has taken place in its bulk, to form the greater part of the limb. In this state it feels hard, but when pressed between the fingers it is readily broken down into small fragments, from which there oozes out in great abundance a sero-purulent, purulent, and sanguineous fluid. This state of the cellular tissue may be regarded as constituting the second stage of the disease, or a state of gangrene. When we arrive at the original seat of the inflammation, none of the physical characters of this pathological state are to be seen. The congestion has disappeared, the blood has been transformed into pus, and the cellular tissue and bloodvessels, converted into a soft spongy substance of various colours, are partly detached in irregular masses, and float in the putrid contents of a ragged excavation. Under these latter circumstances, the muscles, tendons, large bloodvessels, and nerves are often laid bare to a great extent.

Such are the morbid appearances presented by the cellular tissue in fatal cases of erysipelas phlegmonodes, the primary and almost exclusive seat of which is this tissue. They would seem to prove that the rapidly destructive effects of this form of inflammation depend in great measure on the mechanical influence exercised by the effused fluids on the capillary circulation. These fluids must compress the neighbouring veins to a degree that will prevent the return of the blood poured into the capillaries; the function of nutrition must then cease to be accomplished, as is proved by the great diminution of cohesion which is observed to have taken place in the cellular tissue in the second stage of the disease; and as there is no tendency towards the formation of coagulable lymph, by the presence of which alone the inflammatory congestion can be arrested, the state of gangrene which this stage indicates must necessarily terminate in sphacelus. It is indeed well known that free mechanical division of the skin and cellular tissue, especially in the first stage of the disease, is the most effectual means that can be employed to arrest its progress,—a mode of treatment, the obvious effect of which is to remove to a greater or less extent the distention of these tissues, and the mechanical cause by which it is produced."

We have paid some attention to the phenomena of diffuse inflammation of the cellular tissue, and we have carefully dissected limbs in which it has prevailed. We agree with Dr. Carswell, in his description of its pathological characters, and we also agree with him in the opinion, that mortification of the tissue is, in some measure, the result of mechanical agency—of the pressure exerted by the effused serum, lymph, and pus. Examination discloses the successive phases of this destructive inflammation—first, increased vascularity of their texture—then effusion of reddish or of yellow serum—then deposition of lymph and pus—and, finally, the cells being distended with these secretions, sloughing and death to an immense extent. The affection of the cutis is secondary in importance, if not consecutive in point of time, to that of the cellular tissue beneath it; and early incisions will relieve the tension of the skin and will often save it, even when performed at a comparatively late period.

It is difficult to determine what precise cause operates, to favour the extension of this inflammation of the cellular tissue—why, in fact, it is diffuse, instead of being circumscribed by the effusion of coagulable lymph. Whatever that cause may really be, it is certain that diffuse inflammation is most prevalent in persons whose constitutions are impaired, and in situations which are not adapted for maintaining a high state of bodily health. Thus, debauched and drunken persons are particularly prone to diffuse inflammation after injuries, and the inhabitants of large cities, and more particularly hospital patients, are more frequently affected than individuals breathing the pure air of the country, or even than patients in private life.

As our object is the pathology, we shall not enter on the symptoms of diffuse inflammation of the cellular tissue—symptoms which consist essentially in local and external characters. But we are anxious to direct the attention of our readers to the fact, that serious and irremediable mischief is extending or has occurred in the cellular texture, at a time when the skin displays little more than dull red discoloration. Woe to the unhappy patient who is under the care of a surgeon unacquainted with the nature of the disease. Early incisions afford the only chance of safety or recovery.

Carbuncle and boil afford examples of another form of cellular inflammation, terminating in mortification. The only essential difference is this —that, in those affections, the inflammation and sloughing are circumscribed, whilst, in the form to which we have referred so fully, it is diffused, and displays no disposition to be bounded by what is denominated the adhesive process. Yet, in carbuncle and boil, we observe an illustration of the important principle—that the cellular tissue dies sooner than the skin, and that early and free division of the latter checks or arrests the process of mortification in both.

In cynanche parotidœa, or mumps, as Dr. Carswell properly remarks, the cellular tissue of the salivary glands is very similarly situated as in carbuncle. It is this tissue which is the seat of the inflammation, congestion, and effusion; but being prevented by the unyielding nature of the glandular tissue of the organ from accommodating itself to the increased quantity of the fluids poured into it, it soon sloughs, sometimes before the glandular tissue has undergone any remarkable change of colour or consistence, and even before suppuration has commenced.

Dr. Carswell does not notice, and probably he is unacquainted with, a rare, but a very fatal form of inflammation of the cellular tissue. It is diffuse inflammation of that division of the tissue which passes between the muscles and into the deeper parts of the body. We have seen three or four instances of this. The cases terminated fatally, and the cellular tissue, in the situations we have mentioned, displayed the same pathological characters described as affecting the cellular tissue beneath the skin.

The submucous cellular tissue is subject to diffuse and circumscribed inflammation, though this seldom proceeds to any great extent.

" The *diffuse* form of the disease is rarely observed except in the pharynx and larynx, either as a primary affection, or in connexion with erysipelas phlegmonodes of the arms, face, or neck. In these situations, the sloughing of the submucous tissue is very limited, and always accompanied by a corresponding state of the mucous membrane which covers it. The effusions of albuminous and puriform fluids which take place at the same time, occasion a great increase of bulk, produce dysphagia, great difficulty of breathing, or complete asphyxia. Hence the frequently fatal termination of the disease before it has passed into gangrene or sphacelus, and which has sometimes been described under the name of serous, albuminous, and purulent œdema of these parts.

The *circumscribed* form of inflammation of the submucous tissue terminating in gangrene and sphacelus seldom occurs as a primary affection. It follows, in general, inflammation of the mucous membrane, but may afterwards proceed to a considerable extent and occasion sloughing of all the other tissues of the organ in which it occurs, but more especially those of the intestinal tube: such is a frequent cause of intestinal perforation, and the fatal peritonitis by which it is followed."

Diffuse inflammation of the cellular tissue external to the mucous membrane of the larynx and the pharynx, deserves more than a passing notice. It is not unfrequent, extremely insidious, and too generally fatal. The patient appears, and is commonly considered, to be labouring under a common sore throat; no alarm is felt, and, much to the surprise of the friends and the physician, grave symptoms of laryngitis unexpectedly set in, and speedily carry off the individual. On examination after death, lymph and pus are found effused into the cellular texture, external to the mucous membrane of the pharynx and the larynx, and similar depositions usually extend into that intervening between the muscles, and even into the general cellular tissue of the neck. We have seen pus effused, in cases of this sort, into the cellular texture external to the œsophagus, and stretching with that tube down the posterior mediastinum to the chest. The patient commonly dies in this, as in other cases of deep-seated suppuration, with symptoms of a typhoid character; and, occasionally, secondary inflammation of the pleura is established immediately before, and accelerates the fatal termination.

"Gangrene and sphacelus of the *subserous* cellular tissue are seldom observed as a consequence of inflammation; but the former state has more often been met with than the latter, and principally in the sub-peritoneal cellular tissue. The great facility with which this tissue is torn in some cases of acute peritonitis, its pulpy softness in some points, and occasionally a certain degree of fœtor, are circumstances which indicate a near approach to, if not an actual state of, gangrene. In the cavity of the pelvis, and in the iliac and lumbar regions, these appearances are more marked and more easily detected. Not only gangrene but sphacelus of the cellular tissue is occasionally found in these regions in consequence of the extension of inflammation induced more especially by chronic and acute affections of the uterus, rectum, and kidneys."

We have seen an example of diffuse inflammation of the cellular tissue intervening between the muscular and serous tunic of the bowels. It occupied nearly the whole length of the intestinal tube, and originated in the vicinity of an ulcer of the rectum, which extended to the muscular coat of the bowel, and which had been irritated by a surgical examination. Inflammation of the cellular tissue immediately external to the peritoneum is a frequent cause of death after lithotomy. For remarks upon this subject we must direct attention to some late numbers of this Journal.

Mortification of the *mucous membranes,* in consequence of inflammation, is not so frequent as was formerly supposed, the older pathologists confounding with it congestion, melanosis, and so forth. Dr. Carswell observes that the mucous membrane of the throat and intestines is more frequently the seat of this disease than that of any other organ. In the former it is occasionally met with to a limited extent in cynanche tonsillaris and pharyngea, and constitutes the distinctive anatomical character of cynanche maligna or angina gangrenosa. In the latter, it follows as a consequence of certain forms of acute enteritis, either when the inflammation affects the mucous tissue itself, its follicular structure, or both at the same time. In either of these situations the mucous and follicular textures are primarily affected, and may be converted into sloughs of considerable extent without the submucous tissue being destroyed. When thus deprived of their vitality, these textures are, at first, of an ash-grey or straw-colour, and may afterwards become brown or black. They are, however, frequently of the colour of the matter with which they are in contact, the fluid part of which is readily imbibed by the soft spongy tissue of the slough. The mucous membrane which surrounds the slough is generally gorged with blood, indicating either a state of great congestion, or gangrene. When, however, the gangrenous inflammation is confined to the glandulæ agminatæ, and when the greater part or the whole of the follicular structure has sloughed, little congestion or inflammatory redness may remain. In this, as in all other cases of the disease, the existence of sphacelus must be determined by the positive signs of colour, consistence, and smell, supported by the negative evidence arising from the absence of any other cause capable of producing such a condition.

Gangrenous inflammation of the mucous membrane of the air-passages, and of the genital and urinary organs is not frequent. In the former it is seldom seen except as a consequence of the extension of a similar disease of the pulmonary tissue, or of the internal surface of tubercular excavations. In the latter, it is principally the result of the direct operation of mechanical causes, such as calculi contained in the bladder and kidneys, the improper use of instruments, pressure on the head of the child, and so forth.

"The physical characters of gangrene and sphacelus of the mucous membrane of the urinary organs in particular, differ in some respects from those presented by similar states of the respiratory or digestive mucous membrane. Mortification of the mucous membrane of the bladder occurs under two forms, the diffuse and circumscribed. In the former the congestive stage is extreme, and often accompanied by hemorrhage, which gives to the mucous membrane a uniform deep-red colour. Along with this state, dark brown or black patches occupy portions of various extent of the mucous membrane, which, as well as the submucous tissue, is easily torn; and lastly, other portions of this membrane are seen partially detached and converted into a soft spongy substance, having a strong gangrenous odour. In the latter, or circumscribed form of mortification of the mucous membrane of the bladder, the state of congestion is also very great, and almost always accompanied by effusion of blood, which, being confined to circumscribed portions of the submucous tissue, produces ecchymoses of various extent. The state of sphacelus is observed to take place in the centre of the ecchymoses, by the mucous membrane being converted into a soft grey or straw-coloured patch. In the mucous membrane of the pelvis of the kidney, where circumscribed gangrene and sphacelus occasionally occur, the congestive stage is marked by great vascularity, without being followed by hemorrhage—a circumstance which may be attributed to the presence of a much smaller quantity of submucous tissue, and the more intimate union between it and the mucous membrane in this situation than in the bladder."

The physical characters of mortification of the mucous membrane of the uterus, appear to Dr. Carswell to merit and require particular notice. The affection is frequently the direct consequence of acute inflammation of the mucous membrane, occurring immediately or soon after delivery. It may be confined to that portion of the surface of the uterus which gave attachment to the placenta, or it may occupy its whole extent.

"The congestive and gangrenous stage of the disease is indicated by a dark-red or deep venous colour, and occasionally by a deep blue or dirty black colour, probably produced by the chemical action of gaseous products on the blood. A similar discoloration, sometimes a greenish, or more frequently a dirty yellowish-grey colour, is observed in the stage of sphacelus, and is always accompanied by a strong gangrenous odour. The sphacelated tissues are soft, spongy, easily torn, and are generally covered with a putrilaginous fluid substance. When this substance has been washed away, the surface is rough and irregular, sometimes from the presence of the remains of the placenta, but more frequently from an exudation of fibrinous matter, which is occasionally found to cover the whole surface from the fundus to the os tincæ. The substance of the uterus is sometimes affected in this manner to a considerable depth, but it is more frequently only softened without having undergone any other remarkable change. The presence of pus in the fibrous structure of the uterus is seldom observed beyond the gangrened or sphacelated surface, but is frequently met with in the veins and lymphatics."

The *serous membranes* may mortify as well as the subserous cellular tissue. The former derive their supply of blood through the medium of the latter, and it is in consequence of that supply being cut off by alterations in the condition of the cellular tissue, that mortification of the former is produced. It is under these circumstances that sloughing of the serous membranes takes place, which is generally followed by a solution of continuity which establishes a preternatural communication between the serous cavity and that of a neighbouring hollow organ.

"The colour of the sphacelated serous membrane is generally of an ash-grey, sometimes ochrey from the presence of bile or blood. It is soft or spongy, and frequently does not present any peculiar smell. Before it separates, the sub-cellular tissue around it is frequently seen injected with fine red capillaries; occasionally, also, all the tunics of the intestine (when this is the situation of the lesion) are pale, and the accidental opening appears as if it had been made by excision. The external border, however, of the opening is smooth, although irregular, whilst on the internal surface of the intestine it is rough or ragged, or presents other marks of being ulcerated: this is the form of perforation which occurs in chronic ulceration of the glandulæ agminatæ."

A dark brown, dark blue, or black colour of the peritoneum, extending over a considerable portion of its surface, has been already shewn by Dr. Carswell, in his fasciculus on melanosis, to depend on causes very different from those which give rise to mortification.

The physical characters of gangrene and sphacelus of the pleura are similar to those of the same affection of the peritoneum. The most frequent cause of sphacelus of the pleura is the presence of tubercles under the membrane; and the cause next in order of frequency is gangrenous inflammation and superficial abscess of the pulmonary tissue.

Dr. Carswell observes, that gangrene and sphacelus of *fibrous membranes* in general, and *tendons*, take place in the same manner as in serous membranes, that is to say, they are observed to slough only when the cellular tissue surrounding them has previously been destroyed. In like manner also the death of *cartilage* and *bone* is effected by previous disease of the perichondrium, periosteum, or medullary membrane. We feel inclined to doubt the absolute correctness of the assertion that fibrous membranes slough *only* when the surrounding cellular tissue has been destroyed. That tissue being the medium through which the blood-vessels of those textures pass, of course its destruction will probably involve their's. But is this exclusively true? Does it necessarily follow that inflammation of the fibrous membrane itself may not end in sloughing and in death? We confess that we entertain some doubts upon the subject.

The *muscular tissue* seldom inflames and mortifies except from external injury, and in connexion with other textures.

Having thus described the physical characters of mortification in most of the simple tissues of the body, Dr. Carswell proceeds to delineate them in the compound pulmonary tissue, that in which it is most frequent, and in which its general characters are well displayed.

"When the *pulmonary tissue* is affected with gangrene, its colour becomes of a deep red, approaching almost to black, whilst its consistence equals that of hepatized lung or liver. When pressed, it breaks down between the fingers, and there oozes out from it blood and a dirty white or greenish fluid of the consistence of milk or treacle, having a very disagreeable odour. When the state of sphacelus is produced, the pulmonary tissue, seen under the pleura, appears sunk beneath the surrounding surface, presents a dirty white, yellowish grey, brown, or greenish colour, and frequently, when extensive, a mottled aspect, in which all these tints are perceived; it feels flaccid and pulpy, and, when cut into, appears as if converted into a putrid sanies, in which shreds of pulmonary tissue and bloodvessels float or lie detached, and which diffuses around the most insupportable odour of sphacelus."

Mortification of the *pulmonary tissue* may take place in several points of the same lung at the same time, when its extent is usually limited; and it may be circumscribed, or not, by adhesive inflammation. In either case, a certain number of the bronchi are laid open, and if the patient survives for some time the broken down tissues are expectorated in the form of a dirty pulp. Occasionally also the pleura is perforated. In some cases in which the sphacelus has been arrested by the adhesive inflammation, portions of pulmonary tissue of various sizes have been found either loose, or attached by obliterated bloodvessels, and floating in the fluid contents of the excavation. Such excavations have never been observed to cicatrize, but assume an ulcerous character.

Dr. Carswell presents a full account of the state of the *vascular system* in mortification. In this, however, we see nothing to detain us. The most important circumstance is the gradual stagnation and coagulation of the blood in the bloodvessels. This characterizes the stage of gangrene, a recoverable stage; and Dr. Carswell succinctly specifies the process of recovery. When it is about to commence, circulation becomes more active all round the circumference of the affected part; the coagulated blood gradually disappears by the separation of its globules, and their transmission into the neighbouring currents; absorption is manifested by the more or less rapid removal of the effused fluids, sensation and motion return, and the part is restored to the healthy state.

In the transition of gangrene into sphacelus, and the separation of the sphacelated part, it is well known that a line of demarcation is formed by the process of ulceration. That process, it should be recollected, does not take place beyond the obliterated vessels, but the coagulation of the blood in them reaches nearer the heart than the line of separation.

"It is also to the obliteration of the bloodvessels alone that immunity from one of the most dangerous consequences of mortification, viz. hemorrhage, is to be ascribed. The presence of coagulable lymph, its organization and union with the parts into which it has been effused, constituting what is called adhesive inflammation, contributes no doubt to prevent hemorrhage during the process of separation of the dead part, or sloughing. But I am disposed to believe that it is the prevention of hemorrhage from the smaller vessels only that is secured by the adhesive inflammation, while that from the larger ones is prevented by the previous coagulation of the blood within them. That it is to the coagulation of the blood in the large vessels of a limb that we must attribute the non-occurrence of hemorrhage after sloughing, is rendered still more evident from what occurs in some cases of extensive and spreading gangrene, of the inferior extremity for example, and to arrest which it is found necessary to have recourse to amputation. The limb is removed, but the large bloodvessels yield little or no blood; they are, in fact, obliterated by firm coagula. There is no adhesive inflammation in such cases, and gangrene and sphacelus succeed to the operation, in consequence of the vessels not having been divided above the point at which they were obliterated.

Such is the state of the bloodvessels which I have found to accompany sphacelus without hemorrhage. When, on the contrary, hemorrhage occurs in this stage of mortification, these vessels are pervious and filled with fluid or imperfectly coagulated blood; and the cellular and other tissues are more or less infiltrated with serosity, sero-sanguinolent, and puriform fluids."

The second species of mortification from cessation of the circulation described by Dr. Carswell is

2, *Mortification from a Mechanical Obstacle to the Circulation of the Blood.* Mortification from this cause may depend on something preventing the access of arterial blood to a limb, or the return of venous blood from it. The first may be occasioned by ligature of the principal artery of a limb; by coagulated blood; organized or unorganized fibrine, occupying the entire caliber of such an artery or its larger branches; by ossification of the walls of these vessels, or their conversion into a solid fibrous or ligamentous tissue. On the other hand, stagnation of the venous blood may depend on obliteration of the veins caused by the pressure of tumors; by accidental products formed in their cellular sheath; by the presence of fibrine or other solid substances derived therefrom, formed within the veins, and either simply lodged within them, or more or less intimately connected with their lining membrane; and, lastly, by diseases of the heart, which greatly obstruct or prevent the return of the venous blood to this organ.

The physical characters of mortification produced by a mechanical obstacle to the venous circulation have this in common in every organ:—an excessive accumulation of blood in the venous system, trunks, branches, and capillaries of the affected part. Our author's description of this form of mortification in the lower extremities, succeeding disease of the heart, is too accurate and altogether too good to be omitted.

"The first local sign that an obstacle exists to the return of the venous blood from the inferior extremities, is manifested by the appearance of slight œdema around the ankles. The serosity gradually accumulates in those parts, spreads from thence throughout the cellular tissue beneath the skin and between the muscles; the feet, and afterwards the legs, thighs, &c. become swollen; the skin assumes a smooth and glossy aspect, feels tense, and sinks into the cellular tissue when pressed, and does not resume its former shape and situation till raised by the return of the serosity beneath it. The colour of the skin, at first natural, becomes pale and waxy, and may continue in this state during the greater part of the course of the disease. When discoloration of the skin is about to take place, it is seen to depend on the presence of subcutaneous veins, which gradually increase in bulk and number, coalesce in several points, and communicate a slightly mottled aspect to the skin, of a dull red or purple colour. On one or more of these points where the congestion is greatest, and where the skin is less yielding, as over the tibia and above the malleoli, phlyctenæ or large bullæ are formed by the effusion of serosity, either alone or mixed with blood, under the cuticle. When these burst, the cutis beneath presents a dark red or brown colour, and very soon is converted into a dirty yellow or ash-grey slough. The separation of the slough is sometimes preceded by an increase of redness in the surrounding cutis, which, from its anatomical characters, and the increase of temperature and pain by which it is accompanied, is obviously of an inflammatory nature. At other times the redness which precedes or accompanies the separation of the dead part is very slight, and is evidently owing to mere venous congestion, occasioned not only by the disease of the heart, but also by the serosity accumulated in the cellular tissue of the limb, which, from the pressure which it occasions, further retards the return of the blood, and aggravates all the symptoms of the disease. It is, indeed, to this secondary obstacle to the return of the venous blood of the limb, that the termination of the disease in mortification is chiefly to be attributed. It is likewise in consequence of the accumulation of serosity beneath the skin that the state of congestion of the venous system of the limb is not at first perceived."

Dr. Carswell proceeds to state, that in these cases the sloughing rarely extends to a greater depth than the cellular tissue, although the slough may seem on its separation to be deeper.

Mortification of internal organs from an obstacle to the return of the venous blood is always limited, and seldom observed except in the lungs, liver, or intestines. It occurs in the lungs when they are hepatized, the veins being in this state compressed or obliterated, and the arteries much diminished in bulk. Such is sometimes the state of the vessels in the indurated walls of tubercular excavations, as well as of those which traverse the septa. A similar state of the bloodvessels, and induration of the pulmonary tissue, may likewise be produced by an extensive deposition of tuberculous matter. Under such circumstances, portions of the indurated pulmonary tissue become deprived of their vitality, and are separated without the supervention of inflammation. Sphacelus of portions of the liver is not very rare in cases where the organ is nearly filled with cancerous tumors, which produce mechanically extensive obliteration of the veins.

" Mortification from a mechanical obstacle to the return of the venous blood, is well exemplified in intus-susception of the intestines. When the superior portion of intestine passes into the inferior, it carries along with it that part of the mesentery to which it is attached. If it does not suffer much compression, the invaginating process may go on to a great extent; but if it is compressed to such a degree that the return of the venous blood is obstructed, this stage of the disease is arrested, on account of the congestion of all the tunics of the invaginated portion. The congestion is not the consequence of inflammation; it is produced by compression, and in the following manner:—when the mesentery is put on the stretch by the descent of the superior into the inferior portion of the intestine, the veins belonging to it are compressed between the walls of both portions, just at the point where the invagination terminates superiorly. If adhesive inflammation takes place at this point, the peritoneal surfaces of both portions become united, and the veins obliterated. As the arteries are much less affected by pressure than the veins, they continue to pour in their blood into the invaginated portion; this fluid accumulates, and produces an extreme degree of congestion of the mucous and submucous coats, giving to them a deep red or almost black colour. In this state, however, the intestine is not deprived of its vitality. It is in a state of gangrene, but not of sphacelus; for its structure is still entire, and it may, when separated and evacuated, present, after having been macerated for some time so as to deprive it of the blood which it contains, the most perfect state of integrity of all its tunics. Occasionally, however, a portion of the whole of the invaginated intestine is found in a state of complete sphacelus, and is passed in the form of irregular spongy masses or shreds of a dirty ash-grey, brown, or black colour."

Two forms of mortification depend on the cessation of the *arterial circulation*. The first results from rupture of the internal and middle coats of a large artery; the second from the presence of accidental products which occasion obliteration both of the trunks and branches of the arteries of a limb. Professor Turner first described the spontaneous rupture of the internal and middle coats of an artery. In one case, in which mortification took place, the internal and middle coats of the popliteal artery were found lacerated and thickened, and its cavity obliterated in several points by coagulated blood, fibrine, and lymph.

The second of the forms of obliteration of the arteries which has been alluded to, consists in the presence of fibrine, fibrous, or osseous substances in their interior. When the quantity of these substances is such as to interrupt the circulation of the blood through the principal arterial trunk or branches of one of the inferior extremities, mortification is almost always the consequence, from the advanced period of life at which this form of the disease generally occurs, and the very unfavourable state of the arteries to the formation of a collateral circulation. To this form of mortification Dr. Carswell thinks that the terms gangrena senilis, idiopathic and dry gangrene should be limited. We do not exactly understand whether Dr. Carswell intends to employ these names indiscriminately as representatives of the same condition. They certainly are not commonly made use of in this manner. Dry gangrene is generally described and known as a peculiar affection, arising from the operation of several causes, but occasionally dependent on the consumption of improper food. On the other hand, the gangrena senilis is not necessarily dry, for we have seen considerable effusion into the subcutaneous cellular tissue in instances of this complaint.

Dr. Carswell's account of the appearances and progress of gangrena senilis is extremely good. But it does not require any notice here, the description being familiar and the malady not uncommon. With Dr. Carswell's observations on the cause of this kind of gangrene, we are fully disposed to agree. So far as we have seen it is always, or almost always dependent on obstruction or obliteration of the cavity of the arteries. We are tempted to extract our author's observations upon this point, and on the statement that gangrena senilis arises from acute inflammation of the arteries.

"In every case," says he "gangrena senilis which I have examined after death, the arteries of the limb were obliterated to such an extent as to interrupt the circulation of the blood. The obstructing cause consisted, in five or six cases, of a fibrous tissue formed either in the walls or cavities of the arteries, and which had converted these vessels into nearly solid cords of ligamentous consistence. This state was traced from the toes more than half way up the leg; it was always connected with ossification of the larger branches and trunks of the thigh and other parts of the body. In other two cases, the obstruction depended on extensive ossification of the principal arteries of the limb; and in several others it was produced by solid fibrine formed around spicula of bone projecting from the internal surface of the arteries. Connecting these states of the arteries with the external appearances of the mortification with which they are accompanied, there cannot, I conceive, remain a doubt that this form of the disease is the immediate consequence of a deficient supply of arterial blood, from a mere mechanical obstacle to the circulation of this fluid. So palpable indeed, and so frequent are the morbid conditions of the arteries which I have described in gangrena senilis, that it is more than surprising that any individual who has had opportunities of investigating this disease, should have attempted to ascribe its origin to inflammation of these vessels. Leaving aside the incontrovertible evidence of the material facts which demonstrate the truth of the position which I have laid down, there are other circumstances which show that inflammation of the arteries cannot be the cause of gangrena senilis such as I have described it. Whether the inflammation which is supposed to give rise to the disease be considered as of an idiopathic or symptomatic kind, is of no import in the decision of the question. For, in the first place, the obstructing cause, viz. fibrous, fibro-cartilaginous, and osseous tissue, could not owe its origin to inflammation in a space of time so short as that which often marks the duration of the disease; and in the second place, the presence of these accidental tissues in the arteries is no proof that inflammation had ever existed in these vessels. Stagnation of the blood from mechanical or physical causes is sufficient to give rise to the formation of these tissues by means of the fibrine of this fluid."

He states without hesitation that the opinions and observations on this subject, contained in the Leçons Orales of M. Dupuytren are equally inconclusive and defective. And he believes that the appearances there described as evidences of inflammation of the arteries giving rise to mortification, might with more propriety be considered as characterizing some of the worst forms of phlebitis.

3. *Mortification from Debility.* In the forms of mortification already described, no diseased condition of the solids or the fluids preceded, or was necessary to the death of the part. But in mortification from debility, the constitution both of fluids and solids must be to some degree altered from the state of health. Yet cessation of the circulation is here, as in the other cases, the immediate cause of the destruction of the part.

" In mortification from debility, a local accumulation of blood constitutes, in general, the first perceptible change in the part which is about to be deprived of its vitality. This may take place from the part being submitted to pressure merely from its own weight or that of the body, from slight friction, puncture, or other similar causes. In some of these cases the blood accumulates, partly from the influence of gravitation, and partly from compression of the veins; as, for example, in mortification of the soft parts covering the sacrum, heels, elbows, &c. of persons recovering from typhoid fevers, and who are left in that state of prostration which precludes the possibility of changing the position of the body. It is, perhaps, still more conspicuous in some patients similarly confined with paraplegia from injury of the spinal cord.

A state of local congestion is also frequently the only change which precedes the sphacelus of the skin to which leeches have been applied, or which has been scarified or punctured, in greatly debilitated scrofulous children. The skin around the leech-bites assumes a dirty purple, livid, or almost black colour; looks sometimes as if it had been injected with ink; presents no previous redness, heat, or pain, and is not swollen except where the blood is accumulated; it drops off in the course sometimes of twenty-four hours, leaving a number of circular openings, which unite and spread by similar succeeding congestions and sloughing of the contiguous skin."

Dr. Carswell refers to the occurrence of mortification in scorbutus, as another striking instance of the influence of general debility in the production of this disease. Portions of the skin often become gorged with blood, die, and slough, without any previous apparent injury. We witnessed, two or three years ago, a remarkable example of the occurrence of mortification from debility. A patient had purpura, and the patches were larger on the lower extremities than on other portions of the body. A physician bled him, without benefit. He bled the patient a second time; and in less than twenty-four hours after this repetition of the depletion, the left foot had become black, and was perfectly dead. It was affected with dry gangrene, and subsequently separated from the leg at the ankle-joint.

Dr. Carswell includes with the forms of mortification from debility alluded to, the gangrene of the cheek which occurs in children, a frightful, and, happily, a rare form of disease. We need only allude to the works of systematic writers for a description of it. We proceed to the second species of mortification portrayed by Dr. Carswell.

### Mortification from the Violent Operation of Mechanical, Chemical, and Physical Agents.

The mechanical agents, Dr. Carswell observes, which occasion mortification are violent blows and contusions; the chemical, powerfully stimulating substances: and the physical, extreme heat and cold. All these agents produce the same ultimate effect in the part of the body which has been submitted to their influence; that is to say, they deprive, to a greater or less extent, such a part of those properties on which its existence depends. But their destructive effects differ in degree, in extent, and even in kind. For in some cases the tissue may be killed at once, and, in others, its vitality may be so partially destroyed, that recovery may or may not ensue.

1. *Mortification from the violent Operation of Mechanical Agents.* This can, of course, have no definite form, depending, as it must do, on the extent of injury inflicted. Inflammation always supervenes, and by it the sphacelus is occasioned.

2. *Mortification from intense Heat.* The application of excessive heat gives rise to a state of inflammation, gangrene, and sphacelus. The phases are extremely rapid, and this rapidity distinguishes gangrene of this description from that which is the usual consequence of inflammation. Yet we do not suppose that Dr. Carswell implies that the latter *must* precede sphacelation; for the extreme heat kills a part at once, with no preliminary stage of any kind. The characteristic appearances of mortification, produced by the agency of heat, are well delineated by the pen of Dr. Carswell.

"The skin is of a yellow, grey, brown, or black colour; dry and hard; sunk below the level of the surrounding surface, and quite insensible. These are sometimes the only appearances which are at first perceived to follow the action of intense heat, and are certain indices of the complete death of the skin to a greater or less depth. The deeper-seated tissues may also be deprived of their vitality, but to what extent cannot be determined by any change which the cutis may have suffered. The inflammatory redness which succeeds to this state appears almost immediately, and indicates by the rapidity of its course and the peculiar colour which it assumes, the extent both of the gangrene and sphacelus, which could not be determined by any previous change of the cutis produced directly by the heat. The extent of the sphacelus may be said to be always increased by the subsequent inflammation; and parts that were only in a state of gangrene are, by means of it, converted into a state of sphacelus. Whether this state of sphacelus may have been produced by the action of the heat or the subsequent inflammation, the limits of the disease are seldom defined before the end of eight or ten days. The dead are then separated from the living parts, and an abundant suppuration takes place from the denuded surface. The solution of continuity is often imperfectly repaired, in consequence of the exuberant production of granulations, which, instead of acquiring the organization of the cutaneous textures, assume that of contractile tissue, which often gives rise to great deformity of the parts with which it is connected."

We have a very few observations to offer on the subject of the physical effects of heat, which, though rather surgical than strictly pathological, may, we hope, be excused, on account of their practical character.

The physical effects of extreme heat on the surface of the body appear to exhibit the following degrees of difference.

*First,* the skin is simply reddened, and displays the ordinary tint of inflammation.

*Secondly,* the cuticle is separated from the cutis, and serum or lymph is effused between them.

*Thirdly,* the cutis is denuded of its cuticle, and the cutis itself is superficially or entirely killed. If only the superficies is destroyed, a thin film of slough is gradually detached, and the deep remaining portion of the cutis furnishes minute and vascular granulations. If the whole cutis is killed, the slough is of greater density, and the granulations from the subcutaneous cellular tissue are of greater size.

*Fourthly,* the textures subjected to heat are more or less deeply and completely converted into dry hard eschar. The parts in this condition resemble brawn in colour and in feel.

Such are the leading physical degrees of alteration of the tissues producible by heat. They deserve and demand serious attention, from the modifications of treatment they require. But this is too extensive a subject for us to enter on, in an article of an exclusively pathological description.

The consideration of mortification from heat, not unnaturally leads to that of mortification from cold.

3. *Mortification from Cold.* As our author remarks, the local and general effects of severe cold are, in many respects, very similar to those produced by intense heat. If the degree of cold be not very great, the circulation and temperature of the skin are increased, as is shewn by this tissue assuming a redder colour, and feeling warmer than before. On the contrary, if the cold be very intense, it may not give rise to any appreciable degree of local excitement; the vitality of the skin and even of the deeper-seated tissues at the same time, may be either greatly reduced or entirely destroyed by the direct operation of this physical agent. There is, however, this difference between the local effects of heat and cold, viz. that the former may produce complete disorganization of the tissues submitted to its action; whereas the latter never produces such a change. Under the operation of the former the local redness rapidly increases, under that of the latter it rapidly diminishes; and in the same manner does the sensibility increase and decrease under the influence of those agents respectively.

We need scarcely say, that the most frequent cause of mortification of a frozen limb, is exposure to natural or artificial heat after the application of the cold.

4. *Mortification from Stimuli.* The stimuli now referred to are such as exert a chemical influence on the tissues with which they come in contact.

The mineral acids are cited as examples of chemical stimuli, which occasion local death, when they are applied to the skin or mucous membranes of the digestive organs. Their local effects are very similar to those produced by excessive heat, nor do we think that it is necessary to refer to them more particularly.

The last division of this fasciculus is dedicated to—

### Mortification, from the Deleterious Influence of Certain Poisons.

The poisonous substances, says Dr. Carswell, which give rise to mortification are either natural or morbid products, derived from the animal and vegetable kingdoms. The former consist in a peculiar healthy secretion of certain animals, vulgarly termed *venom ;* the latter are generated by a state of disease of the animal solids and fluids, and are called *virus.* The deleterious agent formed by the decomposition of animal matter, and by that diseased state of rye which gives rise to mortification, has received no specific appellation.

1. *Mortification from a Deleterious Agent generated in Healthy Animals.* This form of mortification often follows the bite of the *cobra di copella,* the rattlesnake, and viper.

"When the poison of these animals is inserted into the cutaneous and cellular tissues of one of the limbs, the most acute pain is produced, followed by œdema, swelling, and hardness. If there be any redness of the skin around the wound, it is of short duration, and is succeeded by a livid discoloration, which increases in extent, followed by the formation of phlyctenæ, and diminution of temperature of the part. The hard œdematous swelling of the skin and cellular tissue then becomes soft, crepitates when pressed, and a sanious discharge of a fetid odour runs out from the wound. Such are the local effects of the poison when it posseses a degree of virulence sufficient to destroy the vitality of the part. In milder cases, they are limited to a certain degree of œdematous swelling, accompanied by inflammatory redness of the skin, which, being generally greater than in the former case, shews that the occurrence of sphacelus is essentially determined by the primary effects of the poison on the vital properties of the tissues to which it is applied."

2. *Mortification from a deleterious Agent, generated during the Decomposition of Animal Substances, and in Animals in a State of Disease.*

Dr. Carswell first alludes to the circumstance of mortification of internal organs, especially of the lungs and liver, in connexion with and dependent on mortification of some external part. He concludes that some poisonous agent is generated, and taken into the circulation in cases of this nature. But mortification is much less frequent than depositions of pus under these circumstances, and the subject of the purulent deposites is too curious and too extensive to admit of being thus dismissed in a paragraph.

Our author next adverts to hospital gangrene, as another instance of a similar description ; and to the *pustule maligne,* or *charbon.* Some of our readers may not be aware that the latter is believed to originate in horned cattle, among which it sometimes prevails epidemically to a great extent, and that when it occurs in man it is always derived from such animals.

The parts of the body on which it is generally observed are those which are usually uncovered, as the face, neck, breast, and shoulders; the hands and arms, feet and legs, parts in fact which come in contact with the skin, blood, or flesh of the affected animals. That the blood is strongly impregnated with the septic principle generated in this disease, would seem to be established by the fact, that parts of the body on which this fluid has been deposited have soon after been affected with malignant pustule. Similar effects follow its injection into the veins.

Malignant pustule commences in the form of a small vesicle, filled with a somewhat bloody serosity, accompanied with a circumscribed œdematous swelling of the skin and cellular tissue, which soon extends in breadth, followed by an erysipelatous redness of the skin. As the swelling increases, the skin acquires a glossy aspect, and presents here and there small and large phlyctenæ. The redness soon assumes a livid tint; the central portion becomes brown or black, hard and insensible, whilst the surrounding parts are tense and emphysematous. These changes are produced with more or less rapidity, and sometimes spread to a considerable extent, followed by extensive sloughing of the skin and cellular tissue.

The carbuncle of plague presents local characters very similar to those of the malignant pustule. Dr. Carswell concludes by a description of the effects of the use of diseased rye as an article of food, and of its tendency to give rise to one of the worst varieties of mortification. It may not be amiss to notice this form of gangrene, though some of our readers are no doubt familiar with the descriptions of it, contained in works that have previously been published.

The symptoms that accompany mortification, from the use of diseased rye as an article of food, present considerable variety.

"In one series of cases, mortification is attended or preceded by vertigo, drowsiness, and a malignant form of fever; by a sensation of numbness in the legs, which become afterwards painful, slightly swollen, but not inflamed. The skin is cold and livid, and the sphacelus commences in the centre of the limb, and does not reach the skin till some time after. In a second series of cases, the sphacelated parts are dry, livid, or black; these appearances commencing in the toes and extending gradually upwards, sometimes as far as the thighs. In a third series of cases, the disease commences with lassitude and a sensation as of insects creeping under the skin, and without any febrile symptoms. Soon afterwards the extremities become cold, pale, wrinkled, and benumbed, and at last quite insensible, and incapable of motion; afterwards acute pain is felt, referable to the central parts of the limb. There is now fever and headach; pain extending from the hands and feet to the shoulders, legs, and thighs; and lastly the affected parts become dry, shrunk and black, and drop off at the joints. Entire extremities are thus separated from the body without hemorrhage. Lastly, in other cases, the chief symptoms are, at first, spasmodic contractions of the limbs, afterwards great weakness of mind, voracity of appetite, which generally terminate in fatuity, and sphacelus of some of the limbs. The parts most frequently affected with gangrenous ergotism, as it is called, are the inferior extremities."

Dr. Carswell observes that, however obscure the quomodo of the action of the spurred rye may be, it is evident that the process which terminates in mortification is not of an inflammatory character. All the local changes appear to be produced as direct consequences, he thinks, of the diseased grain, acting through the medium of the blood or nervous system, or of both. In several animals that died after having been fed for some time on spurred rye, and had presented several of the symptoms already mentioned, gangrenous spots are said to have been found in the stomach, intestines, and liver.

This concludes the fasciculus before us—one creditable in every respect to its author. We think it the best he has yet offered to the public, and we conceive that it presents a complete, consistent, and very useful account of the forms and modes of mortification. We strongly recommend it to our readers.

Of the plates, we can conscientiously speak in terms of equally high approbation. They are four in number, and contain eighteen figures. Perforation of the intestines and of the pulmonary pleura are represented in the first—gangrena senilis, and mortification of the skin and subjacent cellular tissue, from an obstacle to the return of the venous blood, are delineated with graphic force in the second—the state of the bloodvessels in gangrena senilis, mortification of the intestine from intus-susception, and mortification of the lung from obliteration of the arteries and veins, the result of chronic induration of the pulmonary tissue, with mortification of the same organs, as a secondary consequence of mortification of the lip, form the subjects of the third plate—and the fourth is devoted to mortification of the lung, circumscribed by adhesive inflammation, to mortification of the skin from leech-bites, and to mortification of the lower lip, occasioned by the bite of an insect. Again we recommend this fasciculus to our readers, to whom we are sure that it is calculated to convey much valuable information.

---

We now turn to the work of M. Cruveilhier, to which we have lately dedicated less attention than its merits fairly demand. The subject to which we shall restrict ourselves at present is, diseases of the fœtus, or some of the diseases that affect or curtail fœtal existence. The different observations of M. Cruveilhier, on this interesting point of pathological anatomy are spread, in a desultory manner, through four fasciculi of his valuable work. However considerable may be the advantages of the publication of extensive and expensive volumes in divided parts, those advantages are diminished, though, perhaps, not counterbalanced, by the interruptions of natural connexion, and diffuseness and repetition of detail. Diffuseness, indeed, is the besetting sin of M. Cruveilhier's publication, and, for aught that we can see, the termination of his and of his readers' labours is almost indefinitely distant. But their value is too great to warrant asperity of criticism, and we gladly turn to glean the useful facts from the scattered pages.

The fœtal maladies delineated by the pencil and the pen of our author are diseases of the brain—diseases of the spinal marrow and monstrosities—diseases of the lungs and thymus gland—diseases of the mouth and pharynx—and, lastly, as a sort of caudal appendage, diseases of the placenta. We shall take these various subjects in the order we have mentioned, though such semblance of method, slight as it may be, is not to be discovered in the fasciculi now spread before us.

### Diseases of the Fœtal Brain.

These are noticed in the fifteenth and seventeenth fasciculi. They are apoplexy—hydrocephalus—atrophy of the convolutions of the brain—and absence of the cerebellum. The 37th number of this Journal contains a tolerably full account of these and some other fœtal affections. We shall therefore present a brief description of the complaints that have been noticed in that number, though we will not waive all specific details, in order that the present article may be tolerably complete.

1. *Apoplexy of newly-born Children.* It appears from the researches of M. Cruveilhier at the Maternité, on the cause of death of still-born children, that apoplexy is that cause in a third of those who die during the progress of labour. He has observed this in almost all the cases, usually considered as asphyxia or congenital weakness.

The constant anatomical character of the apoplexy of newly-born children is, an effusion of liquid blood within the cavity of the arachnoid. Most frequently the effusion is limited to the surface of the cerebellum, sometimes it covers at the same time the posterior lobes of the cerebrum, and in some instances the cerebrum and cerebellum are covered with a layer of blood, the source of which it is sometimes difficult to discover. The extravasation rarely occupies the cavity of the ventricles, but M. Cruveilhier has seen three such cases. He has never witnessed extravasation into the substance either of the cerebrum or cerebellum, although it is occasionally intensely injected. In all the cases which he has seen the spinal dura mater was distended with liquid blood, contained both in the cavity of the arachnoid and in the sub-arachnoid cellular tissue.

These effusions are often accompanied with collections of blood under the hairy scalp, and ecchymoses and small collections are frequently met with between the peritoneum and the cranial bones. Once M. Cruveilhier saw coagulum effused along the whole length of the superior longitudinal sinus, between the dura mater and the bone; and on another occasion the pericranium of the two parietal and occipital bones was separated from them by a thick layer of coagulum. Our author observes that this separation may account for those occipital abscesses that we see occur during the first days of life, and in which the bones are laid bare. He attended a child in whom the large part of the occipital bone came away in three fragments; the child lived.

All apoplectic children are not born dead. In a tolerable proportion respiration is established, either spontaneously or in consequence of the exertions of the practitioner, and they live 24 or 48 hours, or even three or four days in a state of weakness, torpor, immobility, and diminished temperature. Probably some survive when the extravasation is not very great. M. Cruveilhier has never observed paralysis.

The cause of this fœtal apoplexy is in many cases inscrutable. So far from its being due to the application of the forceps, M. Cruveilhier believes that this frequently prevents it. In the majority of instances it is probably owing to the length of the labour. But our author has seen it after an ordinary birth, and even after a very rapid delivery, whilst, on the other hand, labour has lasted forty-eight hours or longer without any accident happening to the child. M. Cruveilhier seems to suppose that constriction of the neck by the cord, or by the neck of the uterus and the external soft parts after the issue of the head, or after that of the trunk in cases of turning or feet-presentation, may produce the disease. Compression of the umbilical cord in a case of presentation of the cord, has been a very manifest cause. M. Cruveilhier also supposes that, in many cases, after the escape of the waters, the uterine contractions on the cord may be an efficient agent.

M. Cruveilhier looks on the affection as always produced by mechanical means, and thinks that a speedy termination of the labour would prevent it in the greater number of cases. He has seen retropulsion of the cord, when this presented, practised successfully at the Maternité; and in feet-presentations a speedy application of the forceps, after the exit of the trunk, will save many children.

The cerebral apoplexy is sometimes accompanied with extravasations in the lung, in the thymus, whilst the liver and spleen are gorged with blood, and the gastro-intestinal mucous membrane is much injected. M. Cruveilhier relates seven cases. Two of these (the first and third) are detailed in the number of this Review from which the preceding text is transferred, and to which we have already sufficiently alluded.

2. *Complete Absence of the Cerebellum in a Girl Eleven Years of Age.* Alexandine La Brosse was born in 1820. She was properly formed, but small, and she continued a delicate child, with a great deficiency of intelligence. At the age of seven years she fell under the observation of Dr. Miguel, who remarked extreme debility of the extremities, a want of intelligence, and an incapability of distinct articulation. In January, 1830, she was admitted into the Foundling Hospital. She was then paralytic in the lower limbs, and spoke with difficulty; though nine years and a half old, she appeared no larger nor more developed than a child of six years of age; she was feeble; imbecile in mind; answered questions with hesitation. In January, 1831, she had fallen into a cachectic state, and kept her bed for three months previously, and had scarcely any power of motion in her legs, although their sensibility remained. She was stupid, never spoke, or answered only yes, or no, to the questions that were put to her, and neither expressed feelings of pleasure nor of pain. It was ascertained that she was addicted to masturbation, and that she had suffered from epileptiform convulsions. In March, 1831, she died.

*Dissection.* Passing over particulars not essential to the interest of the case, we are informed that the inferior occipital fossæ were filled with serous fluid—that in place of the cerebellum, was a gelatinous membrane, attached to the medulla oblongata by two membranous and gelatinous peduncles, the right one of which had been torn. Towards these peduncles were found two small masses of white substance, insulated, and about the dimensions of a pea, on one of which was one of the nerves of the fourth pair. The tubercula quadrigemina were sound, and behind and below them was observed the orifice of the canal of Sylvius. There was no fourth ventricle, nor any trace of the pons varolii. The anterior pyramids terminated in the crura cerebri.

There were miliary tubercles in the lungs, and ulcers in the small intestines.

The reflections of M. Cruveilhier are directed to three points :—first, to the question whether the absence of the cerebellum must be deemed congenital, or the consequence of wasting after birth; secondly, to the phenomena attending the case; thirdly, to the opinion entertained by Gall, that the cerebellum is the seat of the amatory instinct.

1. He believes that the cerebellum was congenitally absent, because there was no trace of the transverse fibres of the annular protuberance and peduncles. Now these are the commissure of the cerebellum and subordinate to the existence of the lateral parts; so that if the latter had been formed originally, the former would probably have been formed also. On the other hand, the hemispheres of the cerebellum are found destroyed by morbid changes, without any sensible diminution of the size of the protuberance and peduncles. For this reason, then, M. Cruveilhier believes that the cerebellum was congenitally absent.

2. Of the phenomena of the case, the weakness of the lower limbs, and the imperfect development of the intellect, are the principal that were observed. But that observation was unfortunately defective, and perhaps inaccurate. Little then can be founded on it.

3. The subject of the case was reported to be addicted to masturbation, and the state of the vagina discovered after death bore out the supposition drawn from her conduct. This fact is in contradiction to the theory of Gall, on the functions of the cerebellum, a theory which M. Cruveilhier assures us is abundantly opposed by physiology and pathology.

3. *Atrophy of the Convolutions of the Brain.* This has been previously noticed in this Journal, but we re-introduce the subject in order to render the present article more complete.

Atrophy of the convolutions of the cerebrum may be general or partial, congenital or subsequent to birth. The remarks will be observed to be chiefly applicable to the atrophy of the latter character.

When it takes place prior to the complete development of ossification, and the deficiency of brain is not made up by a liquid, the bones sink in proportionately to the atrophy. If, on the other hand, ossification of the bones is completed, or if, the ossification being imperfect, serum replaces the deficiency of cerebrum, the cranium may maintain its natural volume or even exceed it, as in certain cases of congenital hydrocephalus where scarcely any cerebral matter exists. In some cases the cranial bones, instead of sinking inwards, exhibit an extreme thickness.

Atrophy of the convolutions presents itself under several forms.

1mo. It often consists in a simple diminution of volume.

2do. In other cases it is a sort of shrivelling of the convolutions, which present an unequal and granular surface. Sometimes this shrivelling is accompanied with different shades of colour, evidently dependent on a former effusion of blood. In either case, the serum fills the void between the bones of the cranium and surface of the brain. This serum occupies the subarachnoid cellular tissue, and when the atrophy is very circumscribed it raises the arachnoid, like a serous cyst.

These two kinds of atrophy are often observed in old men whose faculties are impaired; and in old women known at the Saltpetrière by the name of *gâteuses*, because they pass under them both their urine and their stools, are bed-ridden, and usually die of bed sores.

The granular shrivelling of the convolutions very often co-exists with apoplectic atrophy of the same convolutions, and occupies the vicinity of the cellular cicatrices. In this case the surface of the convolutions presents most commonly a yellowish discoloration, attesting the prior existence of a sanguineous collection in the neighbourhood. This shrivelling co-exists also with yellowish points, as small as grains of sand, and with small linear cicatrices cutting the convolutions longitudinally, or transversely. These points are so small, the lines so narrow, that they would escape even an attentive examination.

3tio. There is a third order of atrophy of the convolutions which consists in their transformation into a cellular tissue of a brownish colour. This however is the result of an apoplectic attack, and M. Cruveilhier reserves its more complete description until he arrives at the alterations occurring in the sites of apoplectic extravasation.

4to. There is an atrophy of the convolutions with induration of the cellular tissue, which sometimes becomes as firm as cartilage. This induration is the result of inflammation.

5to. Another form of atrophy results from the loss of substance undergone by the convolutions after the red softening. One or other face of the convolutions, often both, are deprived of the grey substance to a greater or less extent. The limits of the loss of substance are marked by an irregular line. The surface of the convolution so denuded is covered by a cellular membrane, of a yellowish colour, and more or less vascular.

6to. A sixth kind of atrophy consists in the transformation of a portion, or of the whole of one hemisphere, or of two hemispheres, into a membrane of extreme tenuity. Often there remains of the brain only a nucleus, represented by the optic thalamus, and the corpus striatum more or less altered. This lesion is most commonly congenital, but M. Cruveilhier is in possession of some cases which appear to shew that a similar atrophy extended to the whole of one hemisphere has been post-natal.

7to. In a seventh kind of atrophy, each convolution is converted into a serous cyst with transparent walls, giving at first the idea of an hydatid cyst.

M. Cruveilhier adds a case, which we omit.

4. *Hydrocephalus.* M. Cruveilhier's observations upon this head chiefly appertain to the wasting or imperfect development of the brain, attending collections of water in the cranium.

He observes that, of the numerous lesions of development to which the fœtal brain is liable, hydrocephalic effusion into the ventricles is unquestionably the most important. Hydrocephalus, he proceeds, presents numerous varieties, relating, *first*, to the quantity of liquid and volume of the cranium; *secondly*, to the condition of the brain. In the first point of view, the cranium may exhibit a greater volume than natural, one of proper dimensions, or one too small.

Hydrocephalus may be attended with such augmentation of the bulk of the cranium, that delivery is impossible without its perforation, either spontaneous or artificial. M. Cruveilhier has twice seen this occur spontaneously.

The state of the brain itself is very variable. Sometimes it is perfectly sound. Occasionally it is even hypertrophied; and in cases of this description, hydrocephalic children have produced astonishment by the precocity of their intelligence. But from such intelligence to idiotcy the step is brief, and a few spoonsful more of effusion are sufficient to effect the metamorphosis.

Hydrocephalus may be attended with more or less destruction of the brain, or an anencephalic condition of the organ. Sometimes this is limited to the roof of the ventricles and the upper convolutions; sometimes it involves both the roof and floor; most commonly the substance of the brain itself remains uninjured; but sometimes it is destroyed more or less completely, as M. Cruveilhier found to be the case in two still-born children at the Maternité, in whom neither brain nor cerebellum, nor annular protuberance existed, but merely an indurated and shapeless nodule on the basilar groove of the occipital bone.

There are two kinds of the anencephalic condition of brain:—Hydrocephalic anencephaly, and anencephaly with absence of the cranial vault, or rather with depression and malformation of the bones. In both cases, the absence of the cerebrum must result from the same cause, an error of nutrition, the effect of a serous or inflammatory fluxion, directed on the cerebral substance. That hydrocephalic anencephaly is attended with more or less inflammatory action is demonstrated, in the opinion of our author, *first*, by the brownish-yellow discoloration, which attests the morbid process of reparation of the cerebral substance; *secondly*, by the density, occasionally cartilaginous, of that substance itself.

The study of hydrocephalic anencephaly exhibits the point to which the fœtal organization can support the loss of an important vital organ. There can be no question that, after birth, life is compatible with great morbid alterations, provided they gradually occur; but these alterations are not to be compared with those which occur in the fœtal brain and lungs. Life has gone on after birth, with only a few lobules of the latter permeable to the air, and hydrocephalic children have lived days, and even months, with the minutest fractions of the encephalic mass.

M. Cruveilhier observes that the alteration of the cerebral substance, in hydrocephalus, is not proportioned to the quantity of liquid, the latter generally constituting the measure of the dimensions of the head. Frequently the proportion is inversely to this, the most complete anencephaly existing in combination with a very small head. He relates two cases in support of this position, but we have not space for the details.

Infants with small heads are, as may be supposed, not in all cases hydrocephalic. M. Cruveilhier saw a child that lived eighteen months with a head so small, that it was like that of one of the lower animals. The facial angle was as acute as in the dog, and the occipital crest was developed as in brutes; the vertical diameter of the cranium was one inch. The child gave no sign whatever of intelligence; its limbs were continually in motion; it cried to express its wants; and had, from time to time, convulsive movemets, in one of which it finally expired. The cranium was filled with a brain, which differed from a healthy one only in its small dimensions.

We find that it is impossible to comprise in this department the remainder of the present article. Our readers must turn for its continuation to the Periscope, in which it will be found concluded.*

---

* *Vide* p. 172.

